# Predicting 30-day mortality in intensive care unit patients with ischaemic stroke or intracerebral haemorrhage

**DOI:** 10.1097/EJA.0000000000001920

**Published:** 2023-11-14

**Authors:** Mariëlle K. van Valburg, Fabian Termorshuizen, Bart F. Geerts, Wilson F. Abdo, Walter M. van den Bergh, Sylvia Brinkman, Janneke Horn, Walther N.K.A. van Mook, Arjen J.C. Slooter, Marieke J.H. Wermer, Bob Siegerink, M. Sesmu Arbous

**Affiliations:** From the Department of Intensive Care Medicine, University Medical Center Utrecht, Utrecht University, Utrecht (MKvV, AJCS), Department of Anaesthesiology, Intensive Care and Pain Medicine, Amphia Hospital, Breda (MKvV), National Intensive Care Evaluation Foundation, Amsterdam University Medical Center (FT, SB, MSA), Department of Medical Informatics, Amsterdam University Medical Center, Amsterdam (FT, SB), Healthplus.ai BV, Amsterdam (BFG), Department of Intensive Care Medicine, Radboud University Medical Center, Nijmegen (WFA), Department of Critical Care, University Medical Center Groningen, University of Groningen, Groningen (WMvdB), Department of Intensive Care, Amsterdam University Medical Center, Amsterdam (JH), Department of Intensive Care Medicine, and Academy for Postgraduate Training, Maastricht University Medical Center (WNKAvM), School of Health Professions Education, Maastricht University, Maastricht (WNKAvM), the UMC Utrecht Brain Center, University Medical Center Utrecht, Utrecht, the Netherlands (AJCS), Department of Neurology, UZ Brussel and Vrije Universiteit Brussel, Brussels, Belgium (AJCS), Department of Neurology, Leiden University Medical Center, Leiden (MJHW), Department of Neurology, University Medical Center Groningen, University of Groningen, Groningen (MJHW), Department of Clinical Epidemiology, Leiden University Medical Center (BS, MSA), Department of Intensive Care, Leiden University Medical Center, Leiden, the Netherlands (MSA)

## Abstract

**BACKGROUND:**

Stroke patients admitted to an intensive care unit (ICU) follow a particular survival pattern with a high short-term mortality, but if they survive the first 30 days, a relatively favourable subsequent survival is observed.

**OBJECTIVES:**

The development and validation of two prognostic models predicting 30-day mortality for ICU patients with ischaemic stroke and for ICU patients with intracerebral haemorrhage (ICH), analysed separately, based on parameters readily available within 24 h after ICU admission, and with comparison with the existing Acute Physiology and Chronic Health Evaluation IV (APACHE-IV) model.

**DESIGN:**

Observational cohort study.

**SETTING:**

All 85 ICUs participating in the Dutch National Intensive Care Evaluation database.

**PATIENTS:**

All adult patients with ischaemic stroke or ICH admitted to these ICUs between 2010 and 2019.

**MAIN OUTCOME MEASURES:**

Models were developed using logistic regressions and compared with the existing APACHE-IV model. Predictive performance was assessed using ROC curves, calibration plots and Brier scores.

**RESULTS:**

We enrolled 14 303 patients with stroke admitted to ICU: 8422 with ischaemic stroke and 5881 with ICH. Thirty-day mortality was 27% in patients with ischaemic stroke and 41% in patients with ICH. Important factors predicting 30-day mortality in both ischaemic stroke and ICH were age, lowest Glasgow Coma Scale (GCS) score in the first 24 h, acute physiological disturbance (measured using the Acute Physiology Score) and the application of mechanical ventilation. Both prognostic models showed high discrimination with an AUC 0.85 [95% confidence interval (CI), 0.84 to 0.87] for patients with ischaemic stroke and 0.85 (0.83 to 0.86) in ICH. Calibration plots and Brier scores indicated an overall good fit and good predictive performance. The APACHE-IV model predicting 30-day mortality showed similar performance with an AUC of 0.86 (95% CI, 0.85 to 0.87) in ischaemic stroke and 0.87 (0.86 to 0.89) in ICH.

**CONCLUSION:**

We developed and validated two prognostic models for patients with ischaemic stroke and ICH separately with a high discrimination and good calibration to predict 30-day mortality within 24 h after ICU admission.

**TRIAL REGISTRATION:**

Trial registration: Dutch Trial Registry (https://www.trialregister.nl/); identifier: NTR7438.

**KEY POINTS**

- Thirty-day mortality in ICU-admitted stroke patients is 27% in patients with ischaemic stroke and 41% in patients with intracranial haemorrhage.
- Thirty-day mortality in ICU-admitted stroke patients can be predicted with high discrimination and good calibration using parameters readily available within 24 h after ICU admission.
- Important factors predicting 30-day mortality of ICU-admitted stroke patients are age, lowest GCS in the first 24 h, acute physiological disturbances and the application of mechanical ventilation.
- The APACHE-IV model predicting 30-day mortality in patients with a stroke admitted to an ICU performed within a similar range compared with our developed model.

## Introduction

The incidence of stroke and the subsequent mortality have declined over the past decades, yet stroke is still among the top five leading causes of death in many countries. Furthermore, stroke has a tremendous impact on global healthcare as a major cause of functional disability.^1–3^

Cerebral injury associated with stroke can threaten the function of other vital organ systems, leading to respiratory or circulatory complications.^4,5^ As a result, patients may need treatment in the intensive care unit (ICU). This has occurred more often during the last decade^6,7^ with impaired consciousness and subsequent intubation as the main reason for ICU admission.^8^ Stroke encompasses several subtypes of which intracerebral haemorrhage (ICH) and ischaemic stroke and are the most frequent.^9^

Patients admitted to ICU with a stroke follow a particular survival pattern with a high short-term mortality, but if they survive the first 30 days, a relatively favourable subsequent survival is observed compared with several other diseases necessitating ICU admission.^10^ Although functional limitations exceed mortality risk in clinical relevance,^11^ prognostic factors for functional outcome and mortality do correlate in stroke patients admitted to the ICU.^12–15^ Therefore, it is highly relevant to develop a disease-specific model for ICU-admitted stroke patients to be able to predict which patients are most likely to survive the first critical 30 days. Preferably, clinical parameters readily available upon ICU admission or within the first 24 h of ICU admission should be used as possible predictors.

The generic Acute Physiology and Chronic Health Evaluation (APACHE) IV model^16^ is an established and widely used prediction model for in-hospital mortality in ICU-admitted patients using parameters readily available within 24 h after ICU admission. Although it can be seen as the benchmark prediction model in ICU medicine, it is unknown if this generic model is suitable in disease-specific situations with a markedly specific mortality pattern such as stroke. In addition, the end point of the APACHE-IV model (i.e. hospital mortality) does not match the apparent transition from high to low mortality risk around 30 days, as has been shown in the subset of ICU-admitted stroke patients.^10^

We aimed to develop and validate two prognostic models to predict 30-day mortality for ICU patients with either ischaemic stroke or ICH. The two models were developed based on parameters readily available within 24 h after ICU admission, were internally validated and were compared with the existing APACHE-IV model.

## Material and methods

### Study design

Ethical approval for this study (Ethical Committee N° W 18_049#18.067) was provided by the Institutional Review Board (IRB) of the Amsterdam University Medical Center (Chairperson Professor J. Swinkels) on 20 February 2018. According to the IRB, the study was not subject to the Medical Research Involving Human Subjects Act and therefore the necessity to obtain informed consent was waived.

This cohort study was performed in all 85 Dutch ICUs participating in the National Intensive Care Evaluation (NICE) database between 2010 and 2019, comprising over 90% of all Dutch ICUs.^17^ Inclusion criteria were ICU admission due to ischaemic stroke or ICH according to the APACHE-IV classification system^16^ and fulfilling the APACHE-IV inclusion criteria (therefore, excluding ICU readmissions and ICU admission <4 h). Patient survival data were obtained by linking the NICE database to the national medical insurance claim database (Vektis, Zeist, The Netherlands) using a deterministic linkage algorithm.^18^ The only exclusion criterion was a linkage discrepancy between the NICE and Vektis database.

The NICE database is registered according to the Dutch Data Protection Act, and all data collection was performed in accordance with General Data Protection Regulations. The study is registered in the Dutch Trial Register as NTR7438.

### Data collection

Potential determinants recorded within 24 h of ICU admission and available in the NICE database were selected based on literature, clinical experience, and empirical knowledge. All potential determinants are shown in Supplemental Digital Content 1, which demonstrates definitions and data measurement scales of all potential determinants, and whether determinants are incorporated in the APACHE-IV model.

Age, sex, comorbidities (some new and some incorporated in the APACHE-IV model), and acute physiological disturbance quantified by the APACHE-III Acute Physiology Score (APS)^19^ were included. The APACHE-III APS was used without points assigned for Glasgow Coma Scale (GCS) score. In this manner, the influence of the acute physiologic derangement could be assessed separately from the impaired level of consciousness in this neurologically compromised population. The APS without points for GCS, therefore ranges from 0 to 204 points (the full range including points for GCS is 0 to 252). Supplemental Digital Content 2 elaborates on all elements of the APACHE-III APS and demonstrates its calculation.

The GCS score was incorporated by using the lowest GCS score recorded during the first 24 h of ICU admission (see complementary explanation in Supplemental Digital Content 1, which demonstrates all potential determinants). The GCS was *a priori* categorised into three groups [GCS score 3 to 8 (low); 9 to 12 (moderate) and 13 to 15 (high) as adapted from traumatic brain injury literature].^20,21^ Additionally, whether admission took place in a specialised neurosurgical centre was taken into account. Furthermore, the application of mechanical ventilation, vasoactive medication and the occurrence of acute renal failure or confirmed infection within 24 h of ICU admission were incorporated as potential predictors.

### Outcome

The primary outcome was 30-day mortality after ICU admission. Logistic regression models were used to analyse the association between potential determinants and 30-day mortality.

### Models’ development

All models were built separately for both types of stroke. The dataset was evenly split at random to create a training and validation dataset. Model development, including a preliminary descriptive step, was performed in the training data.

First, a descriptive step was taken by dividing all potential determinants into five categories to assess the contribution of different steps in the collection of clinically relevant information. These categories were based on the chronological order in which information is assessed in common clinical practice: age and sex, chronic health condition, level of consciousness using GCS score as specified above, acute physiological disturbance using the APACHE-III APS (without GCS), and treatment and complications (as specified above and in Supplemental Digital Content 1, which demonstrates all potential determinants). All models also included calendar year of ICU admission, time of admission, and type of hospital.

We assessed the results of logistic regression models for each type of stroke (ischaemic stroke and ICH) separately, with increasing numbers of potential determinants, irrespective of statistical significance or other measure of variable selection in a five-step fashion with category 1, 2, 3, 4, and lastly 5. In each model, hereafter called the *descriptive models*, the new category was included together with all potential determinants of the previous categories. Hence, the fifth model incorporated all above mentioned potential determinants, and is hereafter called the *full model*.

Second, the full models for ischaemic stroke and ICH were simplified with backward selection based on the Akaike Information Criterion (AIC),^22^ selecting the best-fit model to predict 30-day mortality with the greatest amount of variation explained while using the fewest possible number of determinants, hereafter called the *simplified model*.

Third, the prediction of the primary outcome was assessed for both types of stroke using the existing APACHE-IV model^16^ (specification is shown in Supplemental Digital Content 1), which includes age, some comorbidities, APACHE-III APS and admission diagnosis.

### Models’ validation and comparison

The predictive performance of the newly derived simplified models was assessed in the validation dataset. Consequently, both simplified models were compared with the APACHE-IV model for each type of stroke separately. Discrimination was measured by Receiver Operating Characteristic (ROC) analyses with the Area Under the Curve (AUC) as performance measure.

Calibration curves were plotted for both simplified models and the APACHE-IV model to assess accuracy between the estimated and observed number of events. Furthermore, the Brier score^23^ (range 0 to 1) was used to assess the overall performance index reflecting both discrimination and calibration of both simplified models, with lower scores indicating better performance. Differences in predictive performance between the simplified and APACHE-IV models were assessed based on differences between corresponding AUCs with 95% confidence interval (95% CI).

### Sensitivity analyses

Our primary outcome could have been influenced by withdrawal of life sustaining therapy (WLST) decisions, which were not registered in this study. In the literature, the use of mortality within 72 h after ICU discharge as a proxy for WLST decisions has been described.^24,25^ Therefore, sensitivity analyses were performed in the subset of patients who survived more than 72 h after ICU discharge in order to assess the potential impact of WLST decisions on determinants of 30-day mortality in our simplified models.

All statistical analyses were performed using statistical environment R, version 3.6.1 (R Foundation for Statistical Computing, Vienna, Austria). The performance and reporting of this study were in accordance with the TRIPOD statement and checklist^26^ (see Supplemental Digital Content 3, which demonstrates the completed TRIPOD checklist).

## Results

**Fig. 1:**
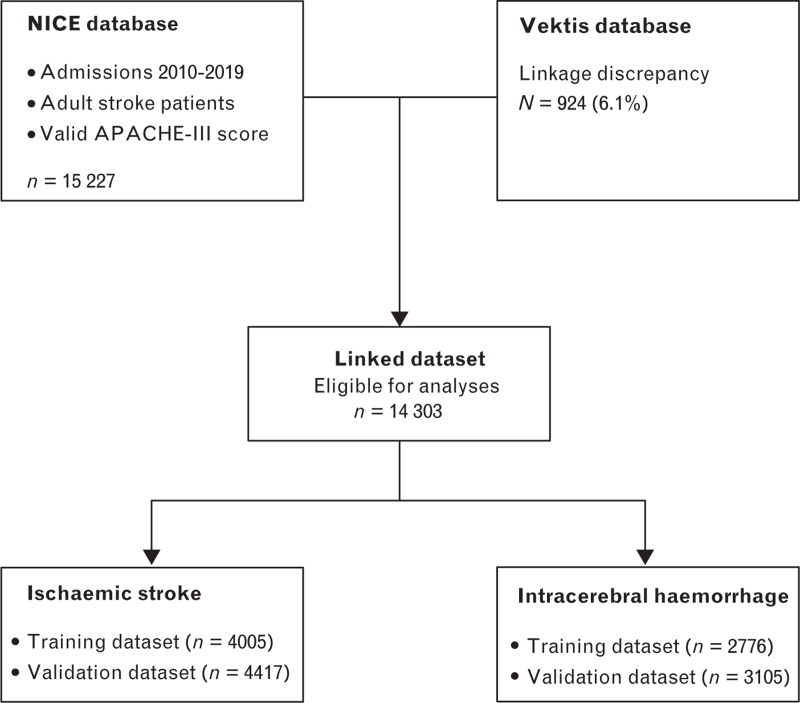
Patient flowchart.

APACHE, Acute Physiology and Chronic Health Evaluation; NICE, National Intensive Care Evaluation.

During the study period (2010 to 2019), 650 815 patients were admitted to the participating ICUs who fulfilled the APACHE-IV criteria and for whom valid record linkage with the Vektis database could be established. The 30-day mortality for all admitted ICU patients after ICU admission was 13%. Of the total ICU population, 14 303 patients (2.2%) were admitted for stroke, 8422 with ischaemic stroke and 5881 with ICH (Fig. 1). Thirty-day mortality after ICU admission was 27% in patients with ischaemic stroke and 41% in patients with ICH. Descriptive characteristics of both patient groups are shown in Table 1.

**Table 1:** Characteristics of all 14 303 patients admitted to the ICU after ischaemic stroke or intracerebral haemorrhage

	Ischaemic stroke			Intracerebral haemorrhage		
Characteristics	Total	Died	30-day survival	Total	Died	30-day survival
*n* (%)	8422	2284 (27.1)	6138 (72.9)	5881	2422 (41.2)	3459 (58.8)
Age (years)	70 [59 to 78]	72 [62 to 79]	69 [58 to 78]	63 [51 to 73]	66 [56 to 75]	60 [49 to 71]
Sex (men, %)	56.5	57.4	56.3	57.3	56.4	57.8
APACHE to III APS (range 0 to 252)	36 [22 to 63]	72 [51 to 90]	28 [20 to 45]	55 [33 to 79]	79 [63 to 92]	39 [26 to 58]
APS without point assigned for GCS score (range 0 to 204)	28 [19 to 40]	39 [28 to 53]	24 [18 to 34]	30 [22 to 41]	37 [26 to 48]	27 [21 to 36]
APACHE III score (range 0 to 299)	51 [35 to 77]	86 [66 to 104]	43 [32 to 59]	67 [44 to 90]	90 [75 to 104]	50 [36 to 68]
APACHE IV probability in %	19 [8 to 48]	58 [36 to 75]	12 [6 to 26]	41 [17 to 69]	69 [51 to 81]	22 [11 to 43]
GCS score (lowest score during first 24 h of ICU admission)	13 [7 to 15]	5 [3 to 10]	15 [11 to 15]	7 [3 to 13]	3 [3 to 6]	11 [7 to 14]
GCS score in groups						
Low (3 to 8)	28.7	66.1	14.8	57.5	86.4	37.3
Moderate (9 to 12)	16.3	17.1	16.0	14.9	6.7	20.7
High (13 to 15)	53.7	16.1	67.7	26.7	5.9	41.3
Not available (%)	1.3	0.7	1.5	0.8	1.0	0.7
Performed neurosurgery in patients with ICH (%)	NA	NA	NA	30.2	23.0	35.3
Specialised neurosurgical centre (yes, %)	33.5	41.8	30.4	75.1	72.7	76.7
Comorbidities (yes, %)						
Chronic respiratory insufficiency or COPD	8.9	11.8	7.8	5.7	6.9	4.8
NYHA class IV	2.9	4.6	2.3	1.8	2.6	1.3
Diabetes	15.1	18.3	13.9	10.2	12.0	8.8
Chronic renal insufficiency or dialysis dependency	3.7	5.6	3.0	2.4	3.4	1.7
Immunologic deficiency or compromised status	2.9	3.6	2.6	3.4	4.1	2.9
Malignancies	2.2	3.5	1.7	3.1	4.3	2.4
ICU treatments or complications within 24 h of ICU admission (yes, %)						
Mechanical ventilation	30.3	61.7	18.6	63.1	82.7	49.4
Use of intravenous vasoactive medication	18.7	35.4	12.5	31.6	41.6	24.6
Acute renal failure	3.5	8.2	1.7	1.8	3.0	1.0
Confirmed infection	8.0	14.7	5.6	3.3	4.3	2.6

Data are presented as: n (%) or median [IQR]. APACHE, Acute Physiology and Chronic Health Evaluation; APS, acute physiology score; COPD, chronic obstructive pulmonary disease; GCS, Glasgow Coma Scale; h, hour; ICU, intensive care unit; IQR, interquartile range; *n*, number; NYHA, New York Heart Association.

### Descriptive models

The descriptive models showed increasing AUCs for every consecutive model (Table 2). The largest increase in discriminative performance occurred with the addition of GCS score in each type of stroke: from AUC 0.64 (95% CI, 0.62 to 0.66) to AUC 0.83 (0.82 to 0.85) in ischaemic stroke, and from AUC 0.62 (0.60 to 0.64) to AUC 0.82 (0.80 to 0.83) in ICH. In the following steps, discriminative performance increased only minimally.

**Table 2:** Descriptive models of five categories for 30-day mortality in patients admitted to an intensive care unit with ischaemic stroke or intracerebral haemorrhage

	Ischaemic stroke	Intracerebral haemorrhage
Prognostic models	AUC (95% CI)	AUC (95% CI)
Category 1: age and sex	0.63 (0.61 to 0.64)	0.61 (0.59 to 0.63)
Category 2: chronic health condition (previous category with add-on comorbidities)^a^	0.64 (0.62 to 0.66)	0.62 (0.60 to 0.64)
Category 3: level of consciousness (previous categories with add-on GCS score, lowest in first 24 h)	0.83 (0.82 to 0.85)	0.82 (0.80 to 0.83)
Category 4: acute physiological disturbance (previous categories with add-on APACHE to III APS)^b^	0.85 (0.84 to 0.86)	0.84 (0.83 to 0.86)
Category 5: complications (previous categories with add-on complications or ICU treatments)^c^	0.86 (0.84 to 0.87)	0.85 (0.83 to 0.86)

APACHE, Acute Physiology and Chronic Health Evaluation; APS, acute physiology score; AUC, area under the curve; CI, confidence interval.

a Comorbidities include chronic respiratory insufficiency/COPD, NYHA class IV, diabetes, chronic renal insufficiency or dialysis dependency, immunologic deficiency or compromised status and malignancies.

b APACHE to III APS was calculated using all parameters except for GCS (as is also shown in Supplemental Digital Content 2), as this was accounted for in category 3 in order to separate acute physiological derangement and impaired level of consciousness. Further explanation is found in the Methods section.

c Complications or ICU treatments include the use of mechanical ventilation or vasoactive medication and occurrence of acute renal failure or confirmed infection. Calendar year of ICU admission (2010 to 2019), time and type of admission and type of hospital are taken into account in all descriptive models.

### Development of simplified models for ischaemic stroke and intracerebral haemorrhage

Table 3 shows the simplified models predicting 30-day mortality in patients admitted to ICU with ischaemic stroke and ICH, respectively. Only eight predictors remained in each model. Important contributors in both models were age, GCS score, acute physiological disturbance (as defined using APACHE-III APS; without GCS), the application of mechanical ventilation, and the occurrence of acute renal failure. Every increasing point in the APACHE-III APS score increased the odds of mortality 1.03-fold (95% CI, 1.03 to 1.04) in patients with both ischaemic stroke and ICH. A GCS score in the lowest category (GCS 3 to 8) increased 30-day mortality risk 14.5-fold (95% CI, 11.5 to 18.3) in patients with ischaemic stroke and 12.6-fold (9.3 to 16.9) in patients with ICH compared with a high GCS score (GCS 13 to 15). Contributors to the simplified models that are not part of the APACHE-IV model, were application of vasoactive medication, the occurrence of acute renal failure and admission to a specialised neurosurgical centre (the latter as a protective predictor and only in patients with ICH).

**Table 3:** Simplified models for predicting 30-day mortality in ICU-admitted patients with ischaemic stroke or intracerebral haemorrhage

	Ischaemic stroke		Intracerebral haemorrhage	
Predictors	OR (95% CI)	*P* value	OR (95% CI)	*P* value
Age^a^ (reference = <45)		<0.0001		<0.0001
45 to 59	1.89 (1.19 to 3.01)		2.13 (1.56 to 2.93)	
60 to 64	2.06 (1.25 to 3.40)		2.52 (1.75 to 3.62)	
65 to 69	2.27 (1.40 to 3.67)		2.88 (2.01 to 4.14)	
70 to 74	2.67 (1.65 to 4.31)		3.94 (2.74 to 5.66)	
75 to 84	3.33 (2.12 to 5.26)		4.96 (3.48 to 7.06)	
≥85	7.12 (4.33 to 11.72)		10.36 (5.84 to 18.40)	
NYHA class IV^b^	2.04 (1.29 to 3.21)	0.002		NA
Diabetes^a^^,^^b^	1.19 (0.94 to 1.51)	0.14		NA
Malignancies^a^^,^^b^		NA	2.73 (1.67 to 4.47)	<0.0001
APACHE to III APS (without GCS)^a^	1.04 (1.03 to 1.04)	<0.0001	1.03 (1.03 to 1.04)	<0.0001
GCS score (lowest during first 24 h)				
High (13 to 15) (Reference)	4.08 (3.21 to 5.19)	<0.0001	2.13 (1.50 to 3.05)	<0.0001
Moderate (9 to 12)	14.51 (11.52 to 18.27)		12.57 (9.34 to 16.92)	
Low (3 to 8)	2.41 (1.01 to 5.74)		10.10 (3.90 to 26.15)	
Not available				
Mechanical ventilation^b^	1.37 (1.12 to 1.68)	0.002	1.69 (1.35 to 2.12)	<0.0001
Use of intravenous vasoactive medication^b^	1.19 (0.95 to 1.47)	0.12	1.32 (1.09 to 1.61)	0.005
Acute renal failure^b^	2.01 (1.29 to 3.13)	0.002	2.26 (1.08 to 4.75)	0.03
Specialised neurosurgical centre^b^		NA	0.50 (0.40 to 0.64)	<0.0001
AIC (full model value)	3345 (3370)		2932 (2952)	
Intercept	−4.8		−4.0	
AUC (95% CI)	0.85 (0.84 to 0.87)		0.85 (0.83 to 0.86)	
Brier score	0.13		0.16	

AIC, Akaike Information Criterion; APACHE, Acute Physiology and Chronic Health Evaluation; APS, acute physiology score; AUC, area under the curve; CI, confidence interval; GCS, Glasgow Coma Scale; ICU, intensive care unit; NA, not admittable, this determinant is listed as it is predictive for the other type of stroke; NYHA, New York Heart Association; OR, odds ratio.

a Predictor is present in the APACHE to IV model. See Supplemental Digital Content 1 for an overview of all parameters incorporated in the APACHE-IV model.^16^

b Reference = no.

### Models’ validation and comparison

**Fig. 2:**
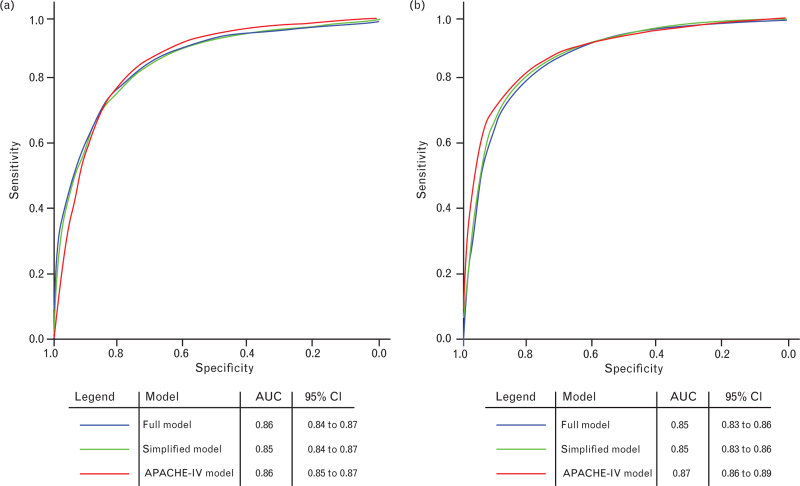
Receiver operating characteristic curves of full, simplified and Acute Physiology and Chronic Health Evaluation-IV models for (a) ischaemic stroke and (b) intracerebral haemorrhage.

APACHE, Acute Physiology and Chronic Health Evaluation; AUC, area under the curve; CI, confidence interval; ROC, receiver operating curve.

ROC curves were constructed for the full models, simplified models and APACHE-IV model for both types of stroke. All models performed with a high accuracy ranging from an AUC of 0.85 to 0.87 (Fig. 2a and b). Both simplified models showed very high discrimination with the AUC = 0.85 (95% CI, 0.84 to 0.87) in ischaemic stroke and AUC = 0.85 (95% CI, 0.83 to 0.86) in ICH. Additionally, both simplified models did not differ in performance from the APACHE-IV model in predicting 30-day mortality with AUC = 0.86 (95% CI, 0.85 to 0.87) in ischaemic stroke and AUC = 0.87 (95% CI, 0.86 to 0.89) in ICH, with overlapping confidence intervals in both types of stroke.

**Fig. 3:**
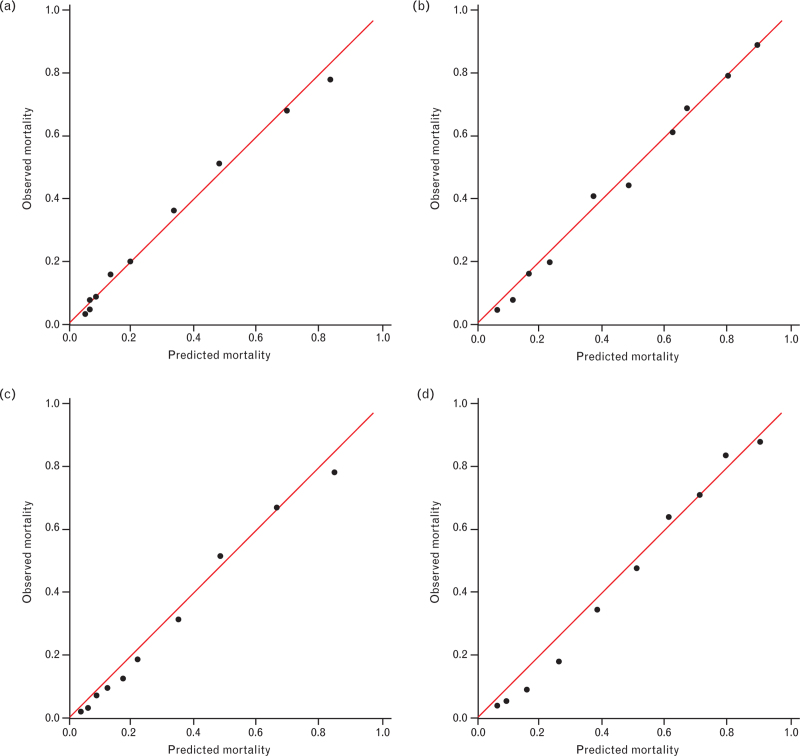
Calibration plots of simplified and Acute Physiology and Chronic Health Evaluation-IV models for ischaemic stroke and intracerebral haemorrhage, respectively.

Calibration plots for both simplified models and APACHE-IV model are shown in Figs. 3a–d. In all plots, observed and expected mortality corresponded very well with the black dots lying closely to the 45° line representing perfect match. Hence, there was an overall good calibration for both simplified models and APACHE-IV model in both types of stroke. Brier scores were 0.13 for the simplified model for ischaemic stroke and 0.16 for ICH, indicating an overall good predictive performance in both simplified models (Table 3).

The plots a–d as shown in this figure represent consecutively (a) the simplified model in ischaemic stroke, (b) the simplified model in ICH, (c) the APACHE-IV model in ischaemic stroke and (d) the APACHE-IV model in ICH for predicting 30-day mortality. The red lines indicate the situation in which predicted mortality perfectly matches observed mortality. The black dots represent the observed probabilities for 10 groups of equal size. APACHE, Acute Physiology and Chronic Health Evaluation; ICH, intracerebral haemorrhage.

### Sensitivity analyses

Sensitivity analyses excluding patients with assumed WLST, occurring in 418 (5.0%) patients with ischaemic stroke and 285 (4.8%) in patients with ICH, demonstrated wider CIs due to smaller groups, but similar effect estimates and model performance in mortality prediction (Supplemental Digital Content 4, which demonstrates the results of the sensitivity analyses).

## Discussion

In this nationwide study, including over 14 000 stroke patients admitted to the ICU, we developed and validated two prognostic models for patients with ischaemic stroke and intracerebral haemorrhage, analysed separately, to predict 30-day mortality after ICU admission with high discrimination and good calibration. Main predictors of 30-day mortality were age, GCS score (lowest during the first 24 h), acute physiological disturbance as described by the APACHE-III APS score, the application of mechanical ventilation and the occurrence of acute renal failure. The newly derived models did not differ in performance from the APACHE-IV model in predicting 30-day mortality. Major new contributors compared with the APACHE-IV model were the use of vasoactive medication, and the occurrence of acute renal failure.

The above mentioned main factors for predicting 30-day mortality in our analyses are comparable to earlier research, although previous study sizes were limited.^8,12–15^ Because of the size of our study, we were able to build models for ischaemic stroke and ICH separately. The two models demonstrated overlapping predictors although differences were found, mainly in effects of comorbidities. Furthermore, the prognostic factors for 30-day mortality found in this study coincide with those in the literature for functional outcome in ICU-admitted stroke patients with age, GCS, the use of mechanical ventilation and acute physiological disturbance (measured using different scoring systems) as main factors.^12–15,27^

The strengths of this study are the large number of patients in a multicentre study design and the comparison to the existing APACHE-IV model, which also has been validated in the Dutch ICU population.^28^ Furthermore, we limited the candidate predictors to determinants present within 24 h of ICU admission and we chose a literature-based endpoint of 30-day mortality taking the specific mortality pattern of ICU-admitted stroke patients into consideration. Additionally, in accordance with the Joint Statement regarding Prognostication in Neurocritical Care, not only did we include admission data, but also data from the subsequent 24 h, and we could incorporate many comorbidities and complications, which is often lacking in prognostic tools for mainly patients with ICH.^29^

Additionally, by design, we incorporated acute physiological derangement by using the well known APS, but eliminated the points assigned for GCS score to separate acute physiological derangement from impaired level of consciousness. Using this approach, we could assess impaired level of consciousness as a separate step in the preliminary descriptive models. Moreover, by taking GCS score into account using the lowest value in the first 24 h of ICU admission, not only did we incorporate a single moment (such as on admission), but created a more robust view of the patients’ level of consciousness during the first 24 h of ICU admission.

Some limitations need to be addressed. Firstly, our results pertain to the subset of acute stroke patients admitted to the ICU. No extrapolation is possible to patients with ischaemic stroke or ICH admitted from the emergency department to the ward or stroke unit. Furthermore, albeit a long and recent accrual period, resulting in a large cohort of over 14 000 ICU-admitted stroke patients, treatment modalities for mainly ischaemic stroke have changed during the study period in which the use of early endovascular thrombectomy has become the standard of care for patients with intracranial large-vessel occlusion in the anterior circulation.^30–36^ This can lead to increased numbers of ICU-admitted stroke patients in the future^6,7^ but would not affect the validity of our study results.

Secondly, the administrative NICE database creates the possibility of analysing prognostic factors for 30-day mortality in a large and extensive cohort of ICU-admitted stroke patients, but unfortunately does not contain data concerning functional outcome. Therefore, this study pertains only to mortality risk of ICU-admitted stroke patients and not to their functional independence or quality of life. This certainly warrants more research with longer follow-up using the above mentioned outcome measurements. It also restrains our ability to implement this new prognostic model in clinical decision-making, especially with regards to counselling families on continued ICU care versus WLST. Unfortunately, we could not collect data concerning National Institutes of Health Stroke Scale (NIHSS) scores^37,38^ or clinical neurological examination, ICH score,^39^ information on affected vascular territories, subtype of stroke, or performed neurosurgical interventions as possible predictors via the NICE database. The latter would have been particularly interesting as we found that having been admitted to a specialised neurosurgical centre was protective, but we did not have specific information on type of neurosurgery performed nor on the decision-making process pertaining to a neurosurgical intervention. Preferably, this should be subject of future studies. We expect that with this crucial stroke-specific information, the likelihood will increase that our model might outperform the already decent performing APACHE-IV model.

Third, all-cause mortality was used as outcome measure, and, therefore, not taking reason of death into account including WLST decisions. Unfortunately, due to legal data protection regulations, we were not able to assess the exact proportion of WLST decisions by reviewing each individual patient report. Insight in this proportion would have improved international generalisability. An increasing shift in European ICUs towards WLST practices has been shown.^40^ Recent literature stated that prediction models in acute brain injury in general are not suited for WLST decision-making^29,41^ as WLST decisions may influence the results in prognostication for severely ill patients such as ICU-admitted stroke patients.^24,25,42,43^ Taking all this into account, we attempted to incorporate this important subject by performing sensitivity analyses, excluding the subset of patients in which WLST decisions were made based on a literature-based definition. These sensitivity analyses demonstrated similar effect estimates for 30-day mortality prediction.

Lastly, with the development of the simplified prognostic models, we also aimed to explore whether using fewer parameters would enable an improvement in clinical bedside use compared with, for example, the APACHE-IV model. Only eight predictors were incorporated in each simplified model. Unfortunately, the incorporation of the APACHE-III APS as a determinant, hampers practical bedside use, as the complexity of the APS still requires computational capabilities.

However, the descriptive models in this study demonstrated that the inclusion of age, sex, six comorbidities and the lowest GCS score during the first 24 h of ICU admission as the only predictors returns an AUC of over 0.8 in both types of stroke, implying good discriminatory ability with only these few and easy assessable variables. This information may assist healthcare providers in daily clinical practice in decision-making for stroke patients admitted to ICU. Ongoing exploration of practical prognostication in this population may, therefore, focus on predictors concerning impaired level of consciousness or more applicable implementations of acute physiologic disturbance measures.

## Conclusion

Prognostication in ICU-admitted stroke patients is commonly performed in clinical practice using different approaches such as clinical impressions based on knowledge and experience, as well as empirical prediction tools. We developed and validated prognostic models with a high discrimination and good calibration to predict 30-day mortality within 24 h for patients who were admitted to the ICU because of ischaemic stroke or ICH, analysed separately. Major predictors were age, GCS score, acute physiologic disturbance, the application of mechanical ventilation and the occurrence of acute renal failure. The two models demonstrated overlapping predictors, although differences were found: presence of cardiovascular comorbidities as NYHA class IV or diabetes were predictive in patients with ischaemic stroke, while ICU admission to a specialised neurosurgical centre was protective in patients with ICH.

The APACHE-IV model predicting 30-day mortality showed similar performance in both populations but is quite complex and includes more variables. The descriptive models in this study showed that even with a few and easy assessable predictors (age, sex, six comorbidities, and lowest GCS score during the first 24 h), a good model performance can be achieved. This may help clinicians in their daily work in decision-making for ICU-admitted stroke patients.

Further research should focus on practical bedside prediction modalities with, preferably, functional independence and quality of life as outcome measures while keeping the specific mortality pattern of ICU-admitted stroke patients in mind. Such studies should incorporate information on clinical neurological examination including brainstem function, and subtype of stroke and vascular territories within the stroke populations (such as brainstem infarction *versus* a hemispheric stroke) to assess possible differences in prognosis among the subtypes of ischaemic stroke and ICH. Other factors that might be examined are, the impact of WLST decisions, and organisational aspects (such as whether admission to a specialised neurosurgical centre, which was protective in patients with an ICH in our analyses, might imply a more interventional approach). An ongoing collaboration between neurologists and intensivists may shift the focus of current datasets to clinical severity scores and incorporation of functional status variables to individualise patient care in a multidisciplinary approach.

## Supplementary Material

Supplemental Digital Content

## Supplementary Material

Supplemental Digital Content

## Supplementary Material

Supplemental Digital Content

## Supplementary Material

Supplemental Digital Content

## References

[1] Virani SS Alonso A Benjamin EJ. American Heart Association Council on Epidemiology and Prevention Statistics Committee and Stroke Statistics Subcommittee. Heart disease and stroke statistics—2020 update: a report from the American Heart Association. *Circulation* 2020; 141:e139–e596. 31992061 10.1161/CIR.0000000000000757

[2] Krishnamurthi RV Ikeda T Feigin VL. Global, regional and country-specific burden of ischaemic stroke, intracerebral haemorrhage and subarachnoid haemorrhage: a systematic analysis of the Global Burden of Disease Study 2017. *Neuroepidemiology* 2020; 54:171–179. 32079017 10.1159/000506396

[3] Xu JQ, Murphy SL, Kochanek KD, Arias E. Mortality in the United States, 2018. NCHS Data Brief, no. 355. Published online 2020. 32487294

[4] Kumar S Selim MH Caplan LR. Medical complications after stroke. *Lancet Neurol* 2010; 9:105–118. 20083041 10.1016/S1474-4422(09)70266-2

[5] Hesse K Fulton RL Abdul-Rahim AH. VISTA Collaborators. Characteristic adverse events and their incidence among patients participating in acute ischemic stroke trials. *Stroke* 2014; 45:2677–2682. 25082807 10.1161/STROKEAHA.114.005845

[6] Kirkman MA Citerio G Smith M. The intensive care management of acute ischemic stroke: an overview. *Intensive Care Med* 2014; 40:640–653. 24658914 10.1007/s00134-014-3266-z

[7] Smith M Reddy U Robba C. Acute ischaemic stroke: challenges for the intensivist. *Intensive Care Med* 2019; 45:1177–1189. 31346678 10.1007/s00134-019-05705-y

[8] de Montmollin E Terzi N Dupuis C. OUTCOMEREA Study Group. One-year survival in acute stroke patients requiring mechanical ventilation: a multicenter cohort study. *Ann Intensive Care* 2020; 10:53. 32383104 10.1186/s13613-020-00669-5 PMC7205929

[9] Sacco RL Kasner SE Broderick JP. AHA/ASA Expert Consensus Document: an updated definition of stroke for the 21st century. *Stroke* 2013; 44:2064–2089. 23652265 10.1161/STR.0b013e318296aeca PMC11078537

[10] van Valburg MK Termorshuizen F Brinkman S. Long-term mortality among ICU patients with stroke compared with other critically ill patients. *Crit Care Med* 2020; 48:e876–e883. 32931193 10.1097/CCM.0000000000004492

[11] Robba C Sonneville R Meyfroidt G. Focus on neuro-critical care: combined interventions to improve relevant outcomes. *Intensive Care Med* 2020; 46:1027–1029. 32206844 10.1007/s00134-020-06014-5

[12] Kiphuth IC Schellinger PD Kohrmann M. Predictors for good functional outcome after neurocritical care. *Crit Care* 2010; 14:R136. 20646313 10.1186/cc9192 PMC2945110

[13] Alonso A Ebert AD Kern R. Outcome predictors of acute stroke patients in need of intensive care treatment. *Cerebrovasc Dis* 2015; 40:10–17. 26022716 10.1159/000430871

[14] Sonneville R Gimenez L Labreuche J. What is the prognosis of acute stroke patients requiring ICU admission? *Intensive Care Med* 2017; 43:271–272. 27695893 10.1007/s00134-016-4553-7

[15] van Valburg MK Arbous MS Georgieva M. Clinical predictors of survival and functional outcome of stroke patients admitted to critical care. *Crit Care Med* 2018; 46:1085–1092. 29608513 10.1097/CCM.0000000000003127

[16] Zimmerman JE Kramer AA McNair DS. Acute Physiology and Chronic Health Evaluation (APACHE) IV: hospital mortality assessment for today's critically ill patients. *Crit Care Med* 2006; 34:1297–1310. 16540951 10.1097/01.CCM.0000215112.84523.F0

[17] Nationale Intensive Care Evaluatie (NICE). Dutch National Intensive Care Evaluation (NICE) Foundation. Available at: http://www.stichtingnice.nl. [Accessed 1 June 2020]

[18] Roos LL Wajda A. Record linkage strategies. Part I: estimating information and evaluating approaches. *Methods Inf Med* 1991; 30:117–123. 1857246

[19] Knaus WA Wagner DP Draper EA. The APACHE III prognostic system. Risk prediction of hospital mortality for critically ill hospitalized adults. *Chest* 1991; 100:1619–1636. 1959406 10.1378/chest.100.6.1619

[20] Valadka AB Narayan RK. Emergency room management of the head-injured patient. *Neurotrauma* 120; New York: McGraw-Hill, 1996.

[21] 10th Edition of the Advanced Trauma Life Support® (ATLS®) Student Course Manual. Chicago (IL): American College of Surgeons; 2018.

[22] Akaike H. A new look at the statistical model identification. *IEEE Trans Automat Contr* 1974; 19:716–723.

[23] Harrell FE Jr Lee KL Mark DB. Multivariable prognostic models: issues in developing models, evaluating assumptions and adequacy, and measuring and reducing errors. *Stat Med* 1996; 15:361–387. 8668867 10.1002/(SICI)1097-0258(19960229)15:4<361::AID-SIM168>3.0.CO;2-4

[24] Parry-Jones AR Paley L Bray BD. SSNAP Collaborative Group. Care-limiting decisions in acute stroke and association with survival: analyses of UK national quality register data. *Int J Stroke* 2016; 11:321–331. 26763918 10.1177/1747493015620806

[25] Souter MJ Blissitt PA Blosser S. Recommendations for the critical care management of devastating brain injury: prognostication, psychosocial, and ethical management: a position statement for healthcare professionals from the Neurocritical Care Society. *Neurocrit Care* 2015; 23:4–13. 25894452 10.1007/s12028-015-0137-6

[26] Collins GS Reitsma JB Altman DG Moons KGM. Transparent reporting of a multivariable prediction model for individual prognosis or diagnosis (TRIPOD): the TRIPOD statement. *Ann Intern Med* 2015; 162:55–63. 25560714 10.7326/M14-0697

[27] Drozdowska BA Singh S Quinn TJ. Thinking about the future: a review of prognostic scales used in acute stroke. *Front Neurol* 2019; 10:274. 30949127 10.3389/fneur.2019.00274 PMC6437031

[28] Brinkman S Bakhshi-Raiez F Abu-Hanna A. External validation of Acute Physiology and Chronic Health Evaluation IV in Dutch intensive care units and comparison with Acute Physiology and Chronic Health Evaluation II and Simplified Acute Physiology Score II. *J Crit Care* 2011; 26:105.e11–105.e18. 10.1016/j.jcrc.2010.07.007 20869840

[29] Wartenberg KE Hwang DY Haeusler KG. Gap analysis regarding prognostication in neurocritical care: a joint statement from the German Neurocritical Care Society and the Neurocritical Care Society. *Neurocrit Care* 2019; 31:231–244. 31368059 10.1007/s12028-019-00769-6 PMC6757096

[30] Berkhemer OA Fransen PSS Beumer D. MR CLEAN Investigators. A randomized trial of intraarterial treatment for acute ischemic stroke. *N Engl J Med* 2015; 372:11–20. 25517348 10.1056/NEJMoa1411587

[31] Goyal M Demchuk AM Menon BK. ESCAPE Trial Investigators. Randomized assessment of rapid endovascular treatment of ischemic stroke. *N Engl J Med* 2015; 372:1019–1030. 25671798 10.1056/NEJMoa1414905

[32] Prabhakaran S Ruff I Bernstein RA. Acute stroke intervention: a systematic review. *JAMA* 2015; 313:1451–1462. 25871671 10.1001/jama.2015.3058

[33] Goyal M Menon BK van Zwam WH. HERMES collaborators. Endovascular thrombectomy after large-vessel ischaemic stroke: a meta-analysis of individual patient data from five randomised trials. *Lancet* 2016; 387:1723–1731. 26898852 10.1016/S0140-6736(16)00163-X

[34] Roaldsen MB Jusufovic M Lindekleiv H. Endovascular thrombectomy and intra-arterial interventions for acute ischaemic stroke. *Cochrane Database Syst Rev* 2021; 6:CD007574. 34125952 10.1002/14651858.CD007574.pub3 PMC8203212

[35] Olthuis SGH Pirson FAV Pinckaers FME. Endovascular treatment versus no endovascular treatment after 6-24 h in patients with ischaemic stroke and collateral flow on CT angiography (MR CLEAN-LATE) in the Netherlands: a multicentre, open-label, blinded-endpoint, randomised, controlled, phase 3 trial. *Lancet* 2023; 401:1371–1380. 37003289 10.1016/S0140-6736(23)00575-5

[36] Langezaal LCM Van Der Hoeven EJRJ Mont’Alverne FJA. BASICS Study Group. Endovascular therapy for stroke due to basilar-artery occlusion. *New Engl J Med* 2021; 384:1910–1920. 34010530 10.1056/NEJMoa2030297

[37] Lyden P Brott T Tilley B. *Improved Reliability of the NIH Stroke Scale using video training* 1994; (127):2220–2226. 10.1161/01.str.25.11.2220 7974549

[38] NINDS rt-PA Stroke Study Group. 121495 tissue plasminogen activator for acute ischemic stroke. *N Engl J Med* 1995; 333:1581–1587. 7477192 10.1056/NEJM199512143332401

[39] Hemphill JCH Bonovich DC Besmertis L. The ICH score: a simple, reliable grading scale for intracerebral hemorrhage. *Stroke* 2001; 32:891–897. 11283388 10.1161/01.str.32.4.891

[40] Sprung CL Ricou B Hartog CS. Changes in end-of-life practices in European Intensive Care Units from 1999 to 2016. *JAMA* 2019; 322:1692–1704. 31577037 10.1001/jama.2019.14608 PMC6777263

[41] Geurts M Macleod MR van Thiel GJMW. End-of-life decisions in patients with severe acute brain injury. *Lancet Neurol* 2014; 13:515–524. 24675048 10.1016/S1474-4422(14)70030-4

[42] Stachulski F Siegerink B Bösel J. Dying in the neurointensive care unit after withdrawal of life-sustaining therapy: associations of advance directives and health-care proxies with timing and treatment intensity. *J Intensive Care Med* 2020; 36:451–458. 32089041 10.1177/0885066620906795

[43] Becker KJ Baxter AB Cohen WA. Withdrawal of support in intracerebral hemorrhage may lead to self-fulfilling prophecies. *Neurology* 2001; 56:766L–772. 11274312 10.1212/wnl.56.6.766

