# Supplementary material for: Predicting 30-day mortality in intensive care unit patients with ischaemic stroke or intracerebral haemorrhage
Source: Eur J Anaesthesiol. 2023 Nov 14;41(2):136–45. doi: 10.1097/EJA.0000000000001920 (PMC10763719; doi:10.1097/EJA.0000000000001920)
Supplement: Supplemental Digital Content [file ejanet-41-136-s001.docx]

Supplemental Digital Content

**Supplemental Digital Content 1.** All potential determinants including definitions, data measurement scales, levels of measurement and presence of determinants in the APACHE-IV model

| Potential determinant | Definition | Measurement scale | Range or levels of measurement | Present in APACHE-IV model^a^ |
| --- | --- | --- | --- | --- |
| Age | Age in years on ICU admission | Categorical | <45; 45-59; 60-64; 65-69; 70-74; 75-84; ≥85 | Yes |
| Sex | Genotypic sex of ICU patients | Categorical | Male - Female | Yes |
| Acute Physiology Score (APS) | APS within APACHE III structure | Continuous | 0-252 | Yes |
| APS without point assigned for GCS | APS within APACHE III structure minus the assigned points for GCS score on ICU admission | Continuous | 0-204 | Indirect as part of APS^b^ |
| GCS score (lowest score during first 24h of ICU admission) | Glasgow Coma Scale; lowest score during the first 24 hours of ICU admission; sedative effects were not scored (in that case the last observed GCS was used); GCS<15 was only scored if a real lowered consciousness level was objectfied | Categorical | High (13-15); Moderate (9-12); Low (3-8);  Not available | Only indirect via APS^b^ |
| Specialized neurosurgical centre | Hospitals that are assigned to provide specialised intracranial neurosurgical care and in which post-operative care can be continued in the hospital’s ICU | Categorical | Yes - No | No |
| Type of admission*^c^* | Medical or surgical (whether elective or emergency) reason for ICU admission | Categorical | Medical - Surgical | Yes |
| Time of admission*^c^* | Time of ICU admission, whether this is during day-time hours of shift hours | Categorical | 8:00-18:00h (day-time); 18:00h-8:00h (shift-time) | No |
| Type of hospital*^c^* | All national hospitals are categorised into (1) academic (university medical centres, 8 in total), (2) teaching hospitals (large hospitals providing highly specialised medical care and collaborating in cooperation STZ (“Samenwerkende Topklinische opleidingsZiekenhuizen”), which is based on medical specialist training programmes) and (3) general hospitals | Categorical | Academic – Teaching - General | No |
| Calendar year*^c^* | Year of ICU admission | Categorical | 2010-2019 | No |

| *(table continues)* | | | | |
| --- | --- | --- | --- | --- |
| Potential determinant | Definition | Measurement scale | Range or levels of measurement | Present in APACHE-IV model*^a^* |
| COMORBIDITIES | | | | |
| Chronic respiratory insufficiency or COPD | Chronic restrictive or obstructive lung disease resulting in severe functional disability OR Registered chronic hypoxia or severe pulmonary hypertension (systolic PAP>40 mmHg) or oxygen dependency OR Diagnosis of COPD more than 6 months ago with use of bronchodilators or steroids prescribed for chronic pulmonary disease | Categorical | Yes - No | No |
| NYHA class IV | Inability to carry on any physical activity without discomfort. Symptoms of heart failure at rest. | Categorical | Yes - No | No |
| Diabetes | Diagnosis of diabetes before ICU admission and treated with oral anti-diabetics or insulin | Categorical | Yes - No | Yes |
| Chronic renal insufficiency or  dialysis dependency | Raised serum creatinine larger than 2.0 mg per 100 ml or 177umol/l combined with diagnosis of chronic renal failure before current hospital admission OR Dialysis dependency (haemodialysis or peritoneal dialysis) before current hospital admission | Categorical | Yes - No | Yes; only dialysis dependency |
| Immunologic deficiency or compromised status | Prolonged use of immunosuppressive therapy OR Use of oral or intravenous corticosteroids (more than 5 days 1mg/kg prednisone or more than 20 days 0.1mg/kg prednisone or equivalent) OR Active chemotherapy or radiotherapy within the last year OR Documented humoral or cellular immunodeficiency | Categorical | Yes - No | Yes; AIDS separately |
| Malignancies | Current solid tumour proven by radiological examination or pathological report  OR  Stadium IV neoplasm OR  Hematologic malignancy as malign lymphoma, acute leukaemia or multiple myeloma | Categorical | Yes - No | Yes |
| *(table continues)* | | | | |
| Potential determinant | Definition | Measurement scale | Range or levels of measurement | Present in APACHE-IV model*^a^* |
| COMPLICATIONS AND ICU TREATMENTS | | | | |
| Mechanical ventilation | Use of invasive or non-invasive mechanical ventilator during first 24 hours of ICU admission | Categorical | Yes - No | Yes^b^ |
| Use of intravenous vasoactive medication | Continuous intravenous medication during at least 1 hours during first 24 hours of ICU admission of one of the following groups: - positive inotropes - vasopressors - phosphodiesterase inhibitor | Categorical | Yes - No | No |
| Acute renal failure | Use of renal replacement therapy during first 24 hours of ICU admission OR  serum creatinine larger than 1.5 mg per 100 ml or 133umol/l combined with oliguria (defined as diuresis of 150 ml or less in 8 hours in a row) | Categorical | Yes - No | Only indirect via APS^b^ |
| Confirmed infection | Confirmed infection using cultures and gram stains OR Combination of perioperative or radiographic observation with clinical findings and laboratory results | Categorical | Yes - No | No |

APACHE, Acute Physiology and Chronic Health Evaluation; ICU, intensive care unit; COPD, chronic obstructive pulmonary disease; NYHA, New York Heart Association; GCS, Glasgow Coma Scale
^a^ Additional covariates in the APACHE-IV model are hepatic cirrhosis and admission origin.
^b^ In the APACHE-IV model, the determinants ‘Mechanical ventilation’ and ‘Acute renal failure’ are used in the APACHE-III APS, to choose between two parameters to assign APS points for. The GCS score on admission is used as a parameter in the APACHE-III APS as well (see also **Supplemental Digital Content 2**), although excluded from the APS in the models derived in this study to separate acute physiologic derangement and impaired level of consciousness as explicated in the Methods section.
^c^ Used to adjust for in descriptive models only
