# Supplementary material for: Predicting 30-day mortality in intensive care unit patients with ischaemic stroke or intracerebral haemorrhage
Source: Eur J Anaesthesiol. 2023 Nov 14;41(2):136–45. doi: 10.1097/EJA.0000000000001920 (PMC10763719; doi:10.1097/EJA.0000000000001920)
Supplement: Supplemental Digital Content [file ejanet-41-136-s002.docx]

**Supplemental Digital Content 2.** APACHE-III APS Calculation

| Parameter (explanation) | Units | Value | APS points |
| --- | --- | --- | --- |
| **Pulse**  (value furthest from 75) | Beats per minute | <=39 | 8 |
|  |  | 40-49 | 5 |
|  |  | 50-99 | 0 |
|  |  | 100-109 | 1 |
|  |  | 110-119 | 5 |
|  |  | 120-139 | 7 |
|  |  | 140-154 | 13 |
|  |  | >=155 | 17 |
| **Mean Blood Pressure**  (value furthest from 90) | mmHg | <=39 | 23 |
|  |  | 40-59 | 15 |
|  |  | 60-69 | 7 |
|  |  | 70-79 | 6 |
|  |  | 80-99 | 0 |
|  |  | 100-119 | 4 |
|  |  | 120-129 | 7 |
|  |  | 130-139 | 9 |
|  |  | >=140 | 10 |
| **Temperature**  (value of core temperature furthest from 38; addition of 1 degree Celsius to axillary temperature prior to selecting worst value) | Degrees Celsius | <=32.9 | 20 |
|  |  | 33-33.4 | 16 |
|  |  | 33.5-33.9 | 13 |
|  |  | 34-34.9 | 8 |
|  |  | 35-35.9 | 2 |
|  |  | 36-39.9 | 0 |
|  |  | >=40 | 4 |
| **Respiratory Rate**  (value furthest from 19; for patients who are ventilated; no points are given for respiratory  rates of 6-12) | Breaths per minute | <=5 | 17 |
|  |  | 6-11 | 8 |
|  |  | 12-13 | 7 |
|  |  | 14-24 | 0 |
|  |  | 25-34 | 6 |
|  |  | 35-39 | 9 |
|  |  | 40-49 | 11 |
|  |  | >=50 | 18 |
| *** PaO2**  (only for non-intubated patients or  intubated patients with FiO2 <0.5) | mmHg | <=49 | 15 |
|  |  | 50-69 | 5 |
|  |  | 70-79 | 2 |
|  |  | >=80 | 0 |
| **OR** | | | |
| *** Alveolar-arterial Oxygen Gradient**  (only for intubated patients  with FiO2 >=0.5) | (none) | <100 | 0 |
|  |  | 100-249 | 7 |
|  |  | 250-349 | 9 |
|  |  | 350-499 | 11 |
|  |  | >=500 | 14 |
| **Hematocrit**  (value furthest from 45.5) | % | <=40.9 | 3 |
|  |  | 41-49 | 0 |
|  |  | >=50 | 3 |
| **White Blood Count** | 10^9^ per liter | <1.0 | 19 |
|  |  | 1.0-2.9 | 5 |
|  |  | 3.0-19.9 | 0 |
|  |  | 20-24.9 | 1 |
|  |  | >=25 | 5 |
| *** Creatinine without acute renal failure (ARF)** (ARF is defined as creatinine >=1.5 mg/dl and urine output <410 cc/day and no chronic dialysis) | Mg/dl | <=0.4 | 3 |
|  |  | 0.5-1.4 | 0 |
|  |  | 1.5-1.94 | 4 |
|  |  | >=1.95 | 7 |
| **OR** | | | |
| *** Creatinine with acute renal failure** | Mg/dl | 0-1.4 | 0 |
|  |  | >=1.5 | 10 |
| *(table continues)* | | | |
| Parameter (explanation) | Units | Value | APS points |
| **Urine Output** | Milliliters per day | <=399 | 15 |
| (total for the day) |  | 400-599 | 8 |
|  |  | 600-899 | 7 |
|  |  | 900-1499 | 5 |
|  |  | 1500-1999 | 4 |
|  |  | 2000-3999 | 0 |
|  |  | >=4000 | 1 |
| **Blood Urea Nitrogen** | Mg/dl | <=16.9 | 0 |
| (value furthest from 0) |  | 17-19 | 2 |
|  |  | 20-39 | 7 |
|  |  | 40-79 | 11 |
|  |  | >=80 | 12 |
| **Natrium** | mEq per liter | <=119 | 3 |
| (value furthest from 145.5) |  | 120-134 | 2 |
|  |  | 135-154 | 0 |
|  |  | >=155 | 4 |
| **Albumin** | Gram per liter | <=1.9 | 11 |
| (value furthest from 3.5) |  | 2.0-2.4 | 6 |
|  |  | 2.5-4.4 | 0 |
|  |  | >=4.5 | 4 |
| **Bilirubin** | Mg/dl | <=1.9 | 0 |
| (value furthest from 0) |  | 2.0-2.9 | 5 |
|  |  | 3.0-4.9 | 6 |
|  |  | 5.0-7.9 | 8 |
|  |  | >=8.0 | 16 |
| **Glucose** | Mg/dl | <=39 | 8 |
| (value furthest from 130) |  | 40-59 | 9 |
|  |  | 60-199 | 0 |
|  |  | 200-349 | 3 |
|  |  | >=350 | 5 |
| **Acid-Base Abnormalities** (points based upon pH-pCO­­_2_ relationship) | pH: no units pCO_2_: mmHg | See score below^a^ | |
| **Neurologic Abnormalities** (points based upon all 3 elements of the Glasgow Coma Scale) | no units | See score below^b^ | |

APS, Acute Physiology Score

The APACHE-III APS (with a score range from 0-252) is calculated using 16 parameters; the APACHE III methodology is precisely followed, in which the most deviant value from the entire first day in ICU is used; this is clarified in the explanation below each individual parameter. All models derived in this study were built with APS without point assigned for GCS (with a score range from 0-204) as described in the Methods section to separate acute physiological derangement and impaired level of consciousness. In order to be complete, the total APS is explicated in this table.

* in 2 parameters a different parameter is used in particular patient conditions as specified in the explanation directly below and indicated using an *

^a, b^ Points for acid-base abnormalities and neurological abnormalities are assigned according to the pH-pCO­­_2_ relationship and all 3 elements of the Glasgow Coma Scale respectively; the tables below elaborate how the APACHE-III APS points for each parameter are calculated.

^a^ Acid-Base Abnormalities

|  | pCO2 (mmHg) | | | | | | | | |
| --- | --- | --- | --- | --- | --- | --- | --- | --- | --- |
| pH | <25 | 25-<30 | 30-<35 | 35-<40 | 40-<45 | 45-<50 | 50-<55 | 55-<60 | >=60 |
| <7.15 | 12 | | | | | | 4 | | |
| 7.15-<7.20 |  |  |  |  |  |  |  |  |  |
| 7.20-<7.25 | 9 | | 6 | | 3 | | 2 | | |
| 7.25-<7.30 |  |  |  |  |  |  |  |  |  |
| 7.30-<7.35 |  |  | 0 | | | 1 | | | |
| 7.35-<7.40 | 5 | |  |  |  |  |  |  |  |
| 7.40-<7.45 |  |  |  |  |  |  |  |  |  |
| 7.45-<7.50 |  |  |  | 2 | |  | | | |
| 7.50-<7.55 |  | 3 | | | 12 | | | | |
| 7.55-<7.60 |  |  |  |  |  |  |  |  |  |
| 7.60-<7.65 | 0 |  |  |  |  |  |  |  |  |
| >=7.65 |  |  |  |  |  |  |  |  |  |

^b^ Glasgow Coma Score

(If a patient is anesthetised, under the influence of anaesthesia, or totally paralysed/sedated during the ENTIRE data collection period, attempt to obtain a GCS from the twelve-hour period prior to ICU admission when GCS was able to be assessed. If no assessable GCS is documented during that time period, zero points should be assigned. If unable to determine verbal score, due to intubation status or similar barriers, use clinical judgement and assign Glasgow Verbal Score according to the following scale: alert/oriented = 5; confused = 3; nonresponsive =1)

| If patient's eyes open spontaneously (4) or to painful/verbal stimulation (2,3), use scale: (Shaded areas represent unlikely clinical combinations. Placing a patient in any of these cells should be done after careful confirmation of clinical findings.) | | | | |
| --- | --- | --- | --- | --- |
|  | Verbal | | | |
| Motor | oriented, converses (5) | confused conversation (4) | inappropriate words, incomprehensive sounds (3,2) | no response (1) |
| obeys verbal commands (6) | 0 | 3 | 10 | 15 |
| localises pain (5) | 3 | 8 | 13 | 15 |
| flexion withdrawal/ decorticate rigidity (4,3) | 3 | 13 | 24 | 24 |
| decerebrate rigidity/no response (2,1) | 3 | 13 | 29 | 29 |
| If patient's eyes do not open spontaneously or to painful/verbal stimulation (1), use scale: (Shaded areas represent extremely unlikely clinical combinations, and should not be used. If these combinations are verified in a clinical setting, no prediction should be generated for the patient.) | | | | |
|  | Verbal | | | |
| Motor | oriented, converses (5) | confused conversation (4) | inappropriate words, incomprehensive sounds (3,2) | no response (1) |
| obeys verbal commands (6) |  |  |  | 16 |
| localises pain (5) |  |  |  | 16 |
| flexion withdrawal/decorticate rigidity (4,3) |  |  | 24 | 33 |
| decerebrate rigidity/no response (2,1) |  |  | 29 | 48 |
