# Supplementary material for: Predicting 30-day mortality in intensive care unit patients with ischaemic stroke or intracerebral haemorrhage
Source: Eur J Anaesthesiol. 2023 Nov 14;41(2):136–45. doi: 10.1097/EJA.0000000000001920 (PMC10763719; doi:10.1097/EJA.0000000000001920)
Supplement: Supplemental Digital Content [file ejanet-41-136-s003.docx]

**Supplemental Digital Content 3.** Checklist of items in reporting a study developing and validating a prognostic model according to the TRIPOD statement^26^

| Section | Report & location |
| --- | --- |
| 1. Title | Yes, both target population and predicted outcome are mentioned |
| 1. Abstract | Yes (objectives, participants, sample size, predictors, outcome, results, conclusion) |
| 1. Background and objectives | Yes, in Introduction (medical context, existing models) |
| 1. Sources of data | Yes, in Methods (subheading ‘Study design’) |
| 1. Participants | Yes, in Methods (subheading ‘Study design’) |
| 1. Outcome | Yes, in Methods (subheading ‘Outcome’) |
| 1. Predictors | Yes, in Methods (subheading ‘Data collection’); definitions, data measurement scales and calculations are specified in Supplemental Digital Content 1 & 2 |
| 1. Sample size | Yes, in Results; patient flowchart in Figure 1 |
| 1. Missing data | Yes, in Methods and in Results (in Table 1 specifically for GCS score) |
| 1. Statistical analysis methods | Yes, in Methods (subheadings ‘Model development’ and ‘Model validation’) |
| 1. Risk groups | Not applicable (two models for two diagnoses) |
| 1. Development vs validation | Yes, in Methods (subheadings ‘Models’ development’ and ‘Models’ validation and comparison’) and in Figure 1 |
| 1. Participants | Yes, in Results (in text and in Figure 1); patient characteristics in Table 1 |
| 1. Model development | Yes, in Results (subheadings ‘Descriptive models’ and ‘Development of simplified models’); Unadjusted association between candidate predictors and outcome are not shown. |
| 1. Model specification | Yes, in Results (subheading ‘Models’ validation and comparison’) and Table 3 |
| 1. Model performance | Yes, in Results (subheading ‘Models’ validation and comparison’) in Table 3, in Figure 2a-b, in Figure 3a-d and in Supplemental Digital Content 4 |
| 1. Model updating | Not applicable |
| 1. Limitations | Yes, in Discussion |
| 1. Interpretation | Yes, in Discussion (limitations of the study, comparison to similar studies) |
| 1. Implications | Yes, in Discussion |
| 1. Supplementary information | Supplemental Digital Content 1-4 |
| 1. Funding | Yes, in subheading “Financial support” |
