# Supplementary material for: Predicting 30-day mortality in intensive care unit patients with ischaemic stroke or intracerebral haemorrhage
Source: Eur J Anaesthesiol. 2023 Nov 14;41(2):136–45. doi: 10.1097/EJA.0000000000001920 (PMC10763719; doi:10.1097/EJA.0000000000001920)
Supplement: Supplemental Digital Content [file ejanet-41-136-s004.docx]

**Supplemental Digital Content 4.** Sensitivity analyses: prognostic models for 30-day mortality in the subset of patients who survived more than 72 hours after ICU discharge as proxy for WLST decisions in order to assess the impact of WLST decisions on the derived simplified models in ICU-admitted patients with ischaemic stroke or intracerebral haemorrhage

| Predictors | Ischaemic stroke | | Intracerebral hemorrhage | |
| --- | --- | --- | --- | --- |
|  | OR (95% CI) | P-value | OR (95% CI) | *P*-value |
| Age (Reference = <45)  45-59  60-64  65-69  70-74  75-84  ≥85 | 1.80 (1.10 to 2.94) 2.05 (1.21 to 3.47) 2.08 (1.25 to 3.46) 2.49 (1.50 to 4.13) 3.02 (1.87 to 4.87) 6.40 (3.77 to 10.86) | <0.0001 | 2.06 (1.49 to 2.84) 2.46 (1.70 to 3.57) 2.79 (1.93 to 4.05) 3.50 (2.41 to 5.09) 4.30 (2.98 to 6.19) 8.52 (4.66 to 15.61) | <0.0001 |
| NYHA class IV*^a^* | 2.20 (1.36 to 3.54) | 0.001 | NA | |
| Diabetes*^a,b^* | 1.11 (0.86 to 1.43) | 0.43 | NA | |
| Malignancies*^a,b^* |  | NA | 2.80 (1.70 to 4.62) | <0.0001 |
| APACHE to III APS (without GCS)*^b^* | 1.04 (1.03 to 1.05) | <0.0001 | 1.03 (1.02 to 1.04) | <0.0001 |
| GCS score (lowest during first 24h)  high (13 to 15) (Reference)  moderate (9 to 12)  low (3 to 8)  not available | 3.68 (2.82 to 4.80) 13.28 (10.35 to 17.05) 3.00 (1.25 to 7.20) | <0.0001 | 1.80 (1.23 to 2.62) 11.11 (8.17 to 15.10) 5.57 (1.89 to 16.45) | <0.0001 |
| Mechanical ventilation*^a^* | 1.47 (1.18 to 1.83) | 0.0005 | 1.76 (1.39 to 2.23) | <0.0001 |
| Use of intravenous vasoactive medication*^a^* | 1.24 (0.98 to 0.56) | 0.07 | 1.35 (1.10 to 1.65) | 0.004 |
| Acute renal failure*^a^* | 1.77 (1.11 to 2.81) | 0.02 | 2.32 (1.10 to 4.91) | 0.03 |
| Specialised neurosurgical centre*^a^* | NA | | 0.52 (0.41 to 0.66) | <0.0001 |
| AIC | 2923 | | 2742 | |
| Intercept | -5.0 | | -4.1 | |
| AUC (95% CI) | 0.86 (0.85 to 0.87) | | 0.85 (0.84 to 0.86) | |
| Brier score | 0.12 | | 0.15 | |

OR = odds ratio; CI = confidence interval; NYHA = New York Heart Association; NA = Not admittable, this determinant is listed since it is predictive for the other type of stroke; APACHE = Acute Physiology and Chronic Health Evaluation; APS = acute physiology score; GCS = Glasgow Coma Scale; AIC = Akaike Information Criterion; AUC = area under the curve
Shaded areas indicate that this parameter is only present in the prediction model of the other type of stroke.
 to indicates that the specific parameter was not incorporated in the predictive model anymore in the subset of patients who survived more than 72 hours after ICU discharge as proxy for WLST decisions
^a^ Reference = No
